# New Ways of Dealing with an Old Problem

**Published:** 1920-12-18

**Authors:** 


### New Ways of Dealing with an Old Problem.

THE London County Council Education Committee is extending its arrangements for cleansing dirty school children.

There has been improvement in the standard of cleanliness since the Education Authority commenced its campaign for cleansing the scholars, but the school medical officer has pointed out that there is now a very serious risk that disease, so prevalent in many parts of Europe, may be spread to this country through the medium of lice.

The L.C.C. now classifies children according to standards of cleanliness. The "unclean" are those who are not actually infested with live vermin, but whose hair contains nits which, if not eradicated, will quickly develop into live vermin. Hitherto the application of the Council's scheme of cleansing has involved the same course of treatment for the slight cases as for the bad ones. In each case the parents are given an opportunity of cleansing their own children, and are offered facilities for taking them voluntarily to the cleansing stations if there are no proper means of cleansing available in the home. The scheme has, moreover, provided for the cutting of the hair of the children, where this has been found necessary. Such a practice is very much resented by both parents and children, and the cleansing stations are in consequence unpopular. Serious disturbance and considerable damage to property have quite recently taken place, and the Council's nurses have been subjected to physical violence at the hands of irate parents. There is, further, a very pronounced objection on the part of parents whose children are only slightly infested to sending them to cleansing stations where infested children are treated.

A method, says the Committee, has now been perfected of removing nits from the hair, by means of which it is possible, by the use of a special kind of comb, to cleanse the head thoroughly in one operation, instead of two or even three as previously, and so to make it unnecessary to cut away any of the hair. The Committee, therefore, has come to the conclusion that it would be of great advantage to arrange for cases of slight infestation to be dealt with by this means. At the moment there is serious congestion at the cleansing stations, and so it has been arranged to send the slightly infested cases to minor ailment centres. Five more nurses will be required. Negotiations are proceeding with Borough Councils to secure the following up of cases so that some attention may be paid to the cleanliness of the children's homes.

